# InjectMeAI—Software Module of an Autonomous Injection Humanoid

**DOI:** 10.3390/s22145315

**Published:** 2022-07-15

**Authors:** Kwame Owusu Ampadu, Florian Rokohl, Safdar Mahmood, Marc Reichenbach, Michael Huebner

**Affiliations:** Computer Engineering, Brandenburg University of Technology, Cottbus-Senftenberg, 03046 Cottbus, Germany; florian.rokohl@b-tu.de (F.R.); safdar.mahmood@b-tu.de (S.M.); marc.reichenbach@b-tu.de (M.R.); michael.huebner@b-tu.de (M.H.)

**Keywords:** edge detection, healthcare, machine learning, pandemic, Pepper robot, pose estimation, Python wrapper, social distancing

## Abstract

The recent pandemic outbreak proved social distancing effective in helping curb the spread of SARS-CoV-2 variants along with the wearing of masks and hand gloves in hospitals and assisted living environments. Health delivery personnel having undergone training regarding the handling of patients suffering from Corona infection have been stretched. Administering injections involves unavoidable person to person contact. In this circumstance, the spread of bodily fluids and consequently the Coronavirus become eminent, leading to an upsurge of infection rates among nurses and doctors. This makes enforced home office practices and telepresence through humanoid robots a viable alternative. In providing assistance to further reduce contact with patients during vaccinations, a software module has been designed, developed, and implemented on a Pepper robot that estimates the pose of a patient, identifies an injection spot, and raises an arm to deliver the vaccine dose on a bare shoulder. Implementation was done using the QiSDK in an android integrated development environment with a custom Python wrapper. Tests carried out yielded positive results in under 60 s with an 80% success rate, and exposed some ambient lighting discrepancies. These discrepancies can be solved in the near future, paving a new way for humans to get vaccinated.

## 1. Introduction

Controlling the spread of Coronavirus variants has become a dire need in hospitals where humans with compromised immune systems converge to seek relief, and contact with bare skin is eminent, particularly during the vaccination process. The voluntary participation of the general citizenry in enforcing agreed-upon regulations on hand sanitizing, social distancing, facial masks, and hand gloves use has not entirely halted the pandemic. Instead, the virus keeps resurfacing in one variant or another in different areas. In the heart of Europe, *Deutsches Zentrum für Neurodegenerative Erkrankungen (DZNE)* has coordinated with the *European Center for Disease Prevention and Control (ECDC)* to retard the spread [1,2].

The adoption of home office practices has paved new ways for available technology to permeate daily activities more than ever. Record sales in personal service robots and medical robots attest to this [3]. While analyzing the adoption of social robots during the pandemic in [4], 156 diverse experiences with 66 different robots could be delineated in various applications implemented worldwide. Over 104 applications of social robots in hospitals were identified, and functions such as carrying food to patients were becoming popular as a means of further reducing human-to-patient contact. Robots have also been developed in the last year to perform pandemic tasks [5]. In Tokyo, an autonomous vacuum sweeper was deployed in hotels housing patients with mild COVID-19 symptoms [6]. 

Minimum nurse-to-patient ratio legislations help bolster stress relief among medical staff. One nurse should cater to at most six patients [7]. However, this is not the case in Germany where the ratio could be as high as 10 patients or more to a nurse [8]. Combined with a global pandemic, the ratio gets worse. This could lead to exhaustion and subsequent resignations among healthcare personnel. Different approaches exist to mitigate this problem. However, the most promising is the introduction of robots into the medical field. 

When introduced at the right points, robots can bring massive improvements to the working conditions of nurses and doctors. One advantage when using a robot is its ability to interact with highly infectious people without the risk of getting infected itself. It does not experience boredom from monotonous tasks. It also poses no risk to the supervisory personnel if it gets disinfected after its work. In addition, it can be used in a task where not much human empathy is involved. A medical task that does not involve much human empathy is the vaccination process, during which it is next to impossible to enforce the social distancing rule on medical personnel as close contact is inevitable. One solution is to engage the assistance of humanoid robots. 

The broad aim of this research, therefore, is to replace medical personnel with an autonomous humanoid robot to perform vaccination activities in contagious environments during pandemics. To the best of our knowledge, no such system has been introduced to the medical society. This will be accomplished by attaching an injection system to an autonomous humanoid robot that can walk independently to interact verbally with patients, identify their poses, and deliver injections on their bare shoulders through a needle attached to one robotic finger; however, only if it can be proven that no one is harmed through this process. 

**Figure 1:**
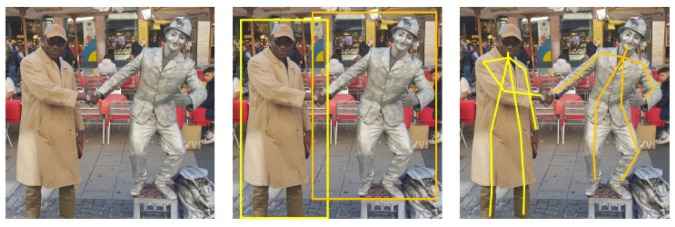
Top-down pose estimation. Input image is cropped for single person pose estimation.

**Figure 2:**
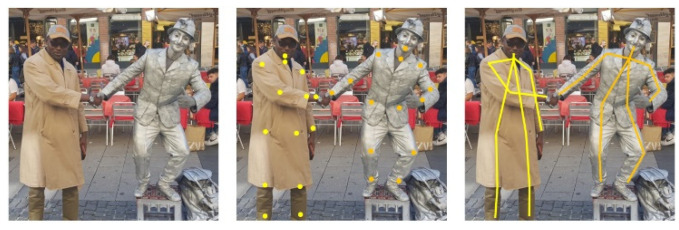
Bottom-up pose estimation. Key points define body parts.

In this first phase of the research, we concentrate on the software aspects of this task. In other words, how to position the robot to be able to deliver the injection to the waiting patient. An overview of this work appeared in [1]. The specific position will be determined through pose estimation. Pose estimation describes the procedure of grouping body parts into person instances. There are different approaches to achieve this. These approaches are dependent on how one considers the problem; either one deals with person instances from which body parts are isolated (Figure 1) or one deals with key points that culminate in person instances (Figure 2). These approaches are known respectively as top-down and bottom-up. 

Accordingly, different objectives can be formulated for accomplishment by a humanoid robot (Pepper). Firstly, it needs to be able to move to patients in the right position. Hence, it needs to identify humans in its surroundings. If a human can be identified, it needs to estimate the pose and decide if the patient is seated for an injection. This is achieved by using pose estimation and classification. If the patient is seated, the injection spot needs to be checked to determine if it is bare (free of clothing) and reachable. These checks are mostly done via images, so the robot needs to transform the image coordinates to actual world coordinates. Without these points, it cannot move to the patient or raise the hand to the right spot. In the end, the robot needs to activate the injection system (hardware) which will be expounded in a separate work. These objectives can then be broken down into the following specific steps:

1. Obtain a 3D orientation of the human relative to the robot.
2. Identify human pose from 2D image.
3. Move the robot next to the seated patient.
4. Find injection position on bare shoulder.
5. Raise the hand of the robot to the required height.

This paper is structured as follows; Section 2 discusses the current state of research involving robots in medical and assisted living environments and compares different pose estimation methodologies. Section 3 explains the rationale behind the mapping of image coordinates to real world coordinates and how the injection point is estimated after patient’s pose classification. Section 4 presents the overall implementation concept from the software perspective, describing the programming ecosystem of Pepper Robot (past and present) and the need for a Python wrapper. Evaluation results are discussed here. Conclusion is provided under Section 5.

## 2. Review of Related Work

The availability of too few nurses for too many patients strongly influences the quality of patient care. Caring for more patients implies spending less time on each individual, which also means interacting less with the patients. In order to interact with more patients, nurses need to do less of other important tasks, work longer, or speed up when doing specific tasks. This may result in worse performance scenarios [9] and/or exhaustion. All over the earth, people are starting to buy more and more robots that can work in the health sector [3,4,5]. Rising sales also portend to increasing usage. The engagement of robots when rightly done enables nurses to deliver better results in a more relaxed atmosphere with less stress.

### 2.1. Robots in Healthcare Assistance

In this direction, Socially Interactive Robots (SIRs) of various shapes and sizes are gaining popularity (Table 1). Paro, a therapeutic baby seal robot, was used to cheer up the elderly [10]. Employed in a mode similar to pet therapy, the robot’s cute appearance and calming effect thrilled a once sleepy audience into excited clusters of chatty elders in a matter of a few weeks, upon its introduction into an experimental care home. The robot gives emotional responses, cuddles with patients, and learns actions that make interactions more delightful, exposing the pleasure of social interactions to people who otherwise find human relationships challenging [11]. An alternative to Paro is Zora, a Nao social robot used for pleasure and entertainment [12]. Zora is more human-like and can move its arms in addition to moving around. A model project to introduce it in the Netherlands resulted in more patients and nurses being entertained; they enjoyed working with it. 

Nurses are faced with time-consuming repetitive tasks such as disinfecting health facilities or drawing blood. Robots can handle these tasks, which do not require much human acuity and only need to be done in the right way. Forgetting to disinfect a spot may lead to dangerous scenarios. For this, the Xenex robot was developed and built. It uses the fact that bacteria and viruses can be eliminated or deactivated with the help of ultraviolet (UV) light and therefore comes equipped with a pulsed xenon lamp. It produces UV lights with a wavelength between 200 and 315 nanometers. With this spectrum, UVA, UVB, and UVC light can be obtained. UVA and UVB are used for eliminating the virus, whereas UVC is used for disabling the virus. This is done by damaging the proteins on the virus’ surface. Afterwards, the virus can no longer attach to human cells, rendering the virus inactive and harmless [13]. To reduce the time spent on drawing blood and analyzing it, the *venipuncture robot* was invented. Veins detection and blood drawing use “*Near-infrared and Ultrasound imaging*”. As for extras, it has microcentrifugation and an optical detection platform that performs the diagnostic analysis [14]. This means that the robot assists the nurses to draw blood–a process that can be dangerous and harmful to patients if the nurses are too stressed or too exhausted. Furthermore, after drawing blood, it automatically performs a blood analysis. This eliminates the extra labor required to check it, meaning the results are obtained faster and without stressing nurses more. 

The problem nurses face is also faced by doctors; there are too few doctors for too many patients. This leads to a very high stress level, which can negatively influence their performance [15]. Wrong decisions or lack of concentration can lead to pretty heavy consequences for patients. To help doctors performing operations in a better and safer way, robotic assistance was introduced into surgery. The most prominent example is DaVinci [16]. It consists of three components: the robotic cart, surgeon console, and endoscopic stack. The cart has four arms, which have multiple degrees of freedom. One arm has the camera, and the other three are designed to hold blunt and sharp trocars. All arms are equipped with a balancing system. A surgeon needs to undergo training to handle the robot. It is primarily used for more minimally invasive surgeries, which are very difficult to perform with conventional techniques [17,18].

**Table 1:** Comparison of Robots in Healthcare Assistance.

Robot	Emotional Support	Injection Applicability
Pepper [1]	Yes	Yes
Paro [10]	Yes	No
Zora [12]	Yes	Less likely
Xenex [13]	No	No
Venepuncture [14]	No	Likely
Davinci [16]	No	Less likely

### 2.2. Two-Dimensional Human Pose Estimation

The pose of a person can be described as a collection of the relative displacements between joints. To generate poses, two different approaches exist that map each person in an image into a collection. Traditional single person pose estimation using deep learning is either derived from a regression method or a parts-detection method [19]. For multi person pose estimation, the first way is to detect all people in a picture and then look for their joints. This is called the top-down approach (Figure 1). The top-down approach is based on looking at an image or scene and classifying the humans in it. It results in bounding boxes that are drawn around spots where a human can be. This is annotated with the confidence score. When the classification confidence score exceeds a pre-defined threshold, the bounding box and the image get piped into body parts detection. The body parts can be easily detected since the number of joints is predetermined. For example, it is not possible that there are two right shoulders in an image when there is only one person in it. The detection algorithm will look for specific attributes that classify the body parts and mark them with their class and confidence score. Again, the body parts that are marked in the result are the ones whose scores lie above a predefined threshold. For example, a key point does not get included if it cannot be scored as a joint mandatory in a person instance. A state-of-the-art representative for this class of algorithms is *Regional Multi-Person Pose Estimation*, also called *AlphaPose* [20]. 

The reverse approach is bottom-up (Figure 2). This is the more favored method, especially for multi-person pose detection. When using the top-down approach, it is necessary to make an early commitment as to which joints are from which person. This can be a big problem in crowded scenes due to occlusion. If the algorithm makes a wrong guess concerning the bounding boxes, it can produce wrong pose estimations. The bottom-up approach at first detects the key points. These key points representing joints are then annotated with the suitable class. The key points that are above a predefined threshold get forwarded to the next stage. This means that all joints are estimated prior to being grouped into person instances. Here, the algorithm will combine the key points into human collections. To combine them, a greedy algorithm is often used. This starts at a point–for example, the nose [21]–and combines the key points that are close to each other to create a person instance. This needs to be done for every joint in the picture until all are combined into a person instance or marked otherwise. 

In [22], G. Papandrou et al. performed a multi-person 2D pose estimation in a unified manner and instance segmentation on images taken “*in the wild*” that employed the bottom-up approach. This is a challenging task as there is no definition as to how much space exists between two persons in an image. Two sequential steps are involved in their box-free approach. First, a single shot convolutional neural network learns to detect key points and predict their relative displacement. This is done by adapting the hybrid classification and regression approach to a multi-person setting resulting in heatmaps. These key points are then grouped into person instances in the second step. When detecting key points, it is difficult to pin-point their specific locations. It is easier and more accurate, in most cases, to assign a score to each key point indicating which key point it is. Thus, each key point is assigned three output channels. The first one contains the heatmap. The heatmaps point to specific key points. Pixels are also marked if they are in a specified radius around the predicted point. The other two channels (offsets) describe the *x* and *y* displacements of the key point. In the next step, the heatmap and offset are combined through *Hough* voting using the Formula (1);

(1) $$h_{k}\left(x\right)=\frac{1}{\pi *R^{2}}*\sum_{i=1:N}p_{k}\left(x_{i}\right)*B\left(x_{i}+S_{i}\left(x_{i}\right)-x\right)$$

where $h_{k}\left(x\right)$ is the *Hough* score, R is radius of the disc used to normalize the errors in the short-range offset to make them commensurate with the heatmap classification loss, $x_{i}$ is a 2D position in the image, $p_{k}\left(x_{i}\right)$ is a predicted heatmap, $S_{i}\left(x_{i}\right)$ is the short-range offset vector, and B(·) denotes the bilinear interpolation kernel. 

The formula yields a result different from zero only when the pixel stays in the range of the current key point. If this is found to be the case, a linear interpolation is carried out between the current pixel (*the pixel for which the Hough score was computed*) and the short-range offset vector for the current pixel. A mid-range offset is used to connect a pair of key points. Connections chosen can be seen in the tree structured kinematic map of a person. The connection prediction task is performed by the CNN, the output of which is given by 2∗(K−1) where K is the total number of key points. The output is further refined twice, using the short-range offset. The mid-range offset does not directly meet the key point but lies within its neighborhood. This means that with the help of the short-range offset we can redirect it onto the right key point. The algorithm can then combine estimated key points into human instances. However, there is no information about which key point is part of which human. Therefore, a fast greedy decoder was proposed. The decoder inserts all scores in descending order into a priority queue. When an element pops out, the algorithm verifies if this key point is in the neighborhood of one that has already been considered, then removes it. If this is not the case, it is seen as a new seed for a new person instance. After this, the learned mid-range displacements are used to combine key points into a kinematic person graph. This is done by greedily connecting pairs of adjacent key points. One major advantage of this approach over other algorithms is that the seed key point is not fixed. This prevents the more accurate face key points from having a high impact on the pose’s score.

The stack hour glass architecture inspired an encoder-decoder top-down network that boosts heat map prediction for all joints in [21]. A second encoder was then used to directly regress the coordinates for all joints in order to circumvent the computation demands associated with large single person heatmaps. By doing so, the heatmap branch could be discarded during inference, resulting in a lightweight architecture suitable for real-time fitness tracking, sign language recognition, and gestural control on mobile devices. In this case, the inference pipeline was split into two; namely, a face detector based on *BlazeFace* and a person (torso) detector. In addition to being a lightning-fast detector that runs in milliseconds, the face detector computes other person-specific alignment parameters such as the midpoint of a person’s hip, a circle circumscribing the person detected, and an inclined line connecting the midpoint of the hips to the midpoint of the shoulders. These initial alignment parameters help in pose tracking. The pose detector is followed by a pose tracker that predicts coordinates, persons, and refines regions of interest in the current frame. 

Cao Z. et al. in [23] used a bottom-up method of non-parametric representation known as Part Affinity Fields to learn to associate body parts with multiple persons in an image. Referred to as OpenPose, the pipeline consists of a feed-forward network that predicts 2D confidence maps for body part location and vectors of Part Affinity Fields (PAF), and a greedy parser that uses the confidence maps and PAF to produce key point coordinates. The system takes a color image and creates a feature map that is fed into a split network of two parallel branches. Branch one creates a confidence map or heat map of body parts. Branch two creates 2D vectors of Part Affinity Fields (PAF), which encode the degree of association between parts, in effect predicting the PAF to preserve location and orientation details. The Convolutional Neural Network (CNN) that is used is initialized with the first 10 layers of the VGG-19 neural network used to generate feature maps for input image classification. These feature maps are then used to predict the first set of the PAF and confidence maps. The following stages refine the prediction from the previous stage using the original image feature map and the $L_{2}$ loss function to punish misclassification. Unlike earlier algorithms, the key points are computed after each stage and not after a fixed number of predictions. The predictions are trained on ground truth confidence maps with annotated key points from COCO, MPII, and a small subset of foot instances from COCO. In confirming that a pair of body parts belong to the same person, a confidence measure is needed. One way is to add a midpoint between each pair of body parts and to check for its occurrence between candidate part detections. Generally, when taking the midpoint between two points, the key points are reduced to one point. This means that the region information is thrown away. 

For PAF, the vector between key point 1 and 2 is used with information about the limb width. This results in a region that describes the connection between the key points. This has a hugely positive effect when working in crowded spaces because the greedy algorithm can check every possible connection and visit the pixels in between to compute the average PAF value. The PAF value is zero if the point is not between the two points and in the given height. Otherwise, it is defined as the length of the vector between the two key points. After the computations, the average value is taken, and longer distance limbs associations are punished. In the last step, all connections are sorted by their average PAF value. 

The work of Bulat A. et al. [24] revisited residual units and introduced a new learnable soft gated skip connection to improve accuracy with limited memory and computational power in a hybrid Hour Glass and U-Net model. Skip connections or the Residual Neural Network (ResNet) made it possible to train very deep neural networks by not simply connecting layer i to layer i+1 but, for example, to layer i+2. This reduces the impact of vanishing gradients and speeds up the training process. The choice of soft gating was informed by the fact that hard gating does not converge at all times in ResNet. Weights are fixed under hard gated connections, whereas they can be learned under soft gated connections through backpropagation. 

The Hour Glass model benefits highly from the connections, but all architectures take it for granted that the skip connections improve the model’s performance and accuracy. The connections are implemented with a learnable value for each input channel and not a single learnable value for all channels. The proposed model consisted of a series of encoders and decoders. Without gated skips, the input image at the first layer of the encoder becomes compromised along with the encoder. The encoder reduces the dimensions of the features and the decoder reverses the reduction. The introduced gated skip connections connect the encoder layer with its counterpart in the decoder as a theoretical second input. Directly adding the features that are coming from different positions in the models seems to be suboptimal. Thus, options were explored along the lines of concatenating features and processing them using 3 × 3 filters or combining them with the aid of grouped convolutional networks.

### 2.3. Deductions

The foregoing approaches help to accomplish different goals: OpenPose addresses pose estimation “*in the wild*”; BlazePose has a lightweight architecture that is tailored to run on less powerful hardware and is used for sports activities tracking and sign language recognition. Soft gated skips also adapt well to limited hardware resources—computational and memory wise.

#### 2.3.1. Hardware

Pepper’s tablet contains an ARM Cortex-A7 [25]. This CPU was released in 2013 and is built for low performance with low power consumption. It has neither a built-in GPU nor an integrated co-processor. It has a theoretical performance of 0.8 GFLOPs for 32-bit floating-point operations [26]. FLOPs describe the number of floating-point operations that are needed to run one instance of a model. Full BlazePose implementation requires 6.9 MFLOPs to complete one calculation, whereas its lite version requires 2.7 MFLOPs. In contrast, the smaller model of soft gated skip connections needs 9.9 GFLOPs, which is more than 1000 times that of the full implementation of BlazePose. The base model of OpenPose needs 160.36 GFLOPs for one single instance run [27].

#### 2.3.2. Accuracy

Soft gated skip connections achieved an accuracy of 92.4% on the MPII dataset [28]. OpenPose had an 88.8% total accuracy [27]. BlazePose does not include any reference to MPII but exhibits a slightly degraded performance than OpenPose on the Augmented Reality (AR) dataset [21]. The AR dataset is an in-house dataset comprising a wide variety of people in the wild.

#### 2.3.3. Adaptability

Another consideration concerns the flexibility of implementation in the Android Integrated Environment. BlazePose has a simple implementation which can be ported with ease [29]. Soft gated skip connection is available as a TensorFlow Model [30]. Although it can be used with the Kotlin programming language, a classifier has to be created for it. Some *github* repositories have tested OpenPose, but there is no easy-to-use implementation.

#### 2.3.4. Speed

The estimation speed is essential for the application to avoid longer patient waiting times. A longer waiting period can lead to loss of confidence in the process. BlazePose excels in terms of accuracy–speed trade off (Table 2).

## 3. Design and Development

**Figure 3:**
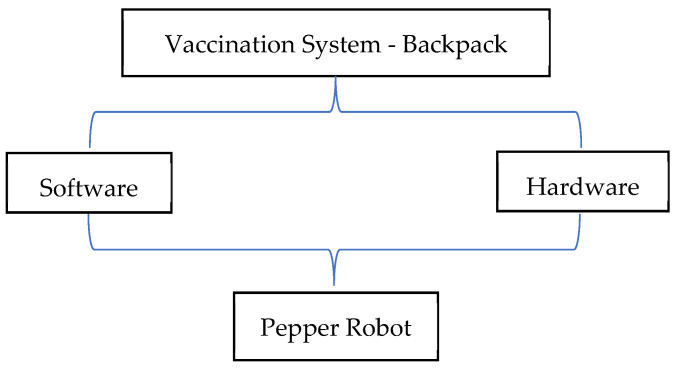
Autonomous Vaccination System Co-design.

**Figure 4:**
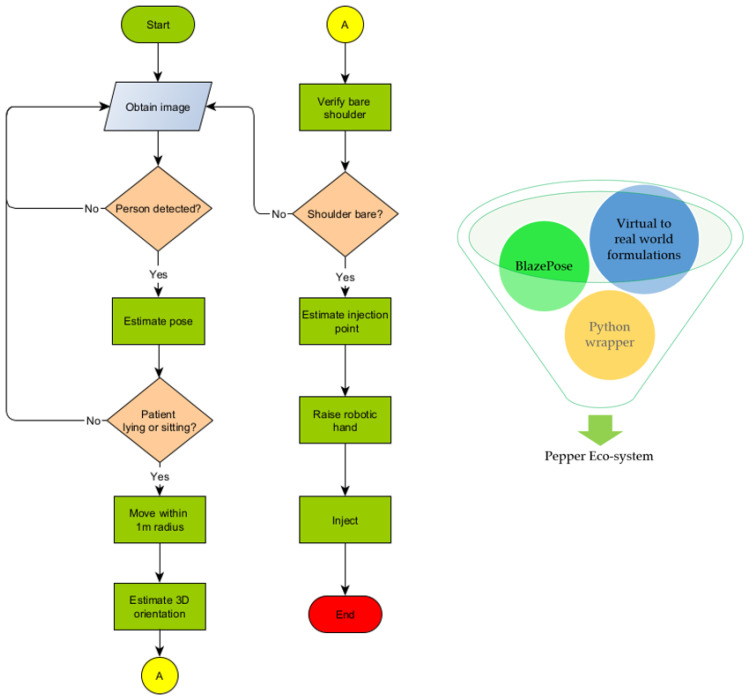
Vaccination Algorithm in relation to Pepper programming Eco-system.

The Pepper humanoid robot has touch sensors, two RGB cameras, and depth sensors in its head that detect human presence to support its emotion engine. In addition, sonar, laser, gyro, and bumper sensors help it to avoid obstacles up to a maximum speed of 3km/h. We take advantage of these sensors to detect human presence and use pose estimation to determine a patient’s preparedness for injection. The robot follows a trajectory to position itself and perform bare shoulder verification for injection point spotting. The robot then raises its hand to the injection point to begin the vaccination process. The setup is as shown in Figure 3. The vaccine will be contained in a backpack to be worn by the Pepper robot. The sequence of steps, described earlier, is depicted in Figure 4 where (A) designates the point of connection between the left and right branches of the flowchart. An image of the algorithms in relation to the Pepper programming ecosystem is also shown.

### 3.1. Bare Shoulder Verification

Our approach is based on the following assumptions;

1. The patient wears a short sleeve shirt that does not cover the whole upper arm but ends above the elbow.
2. The patient does not wear a tattoo of the same color as the shirt and skin.

When the shirt color is different from the skin color, we can predict an edge between the color of the skin and that of the shirt. The color difference helps in computing color gradients. Furthermore, we assume that the detected key points are correct and that the upper arm can be approximated using a line that meets two points on the arm but moved closer to the edge of the arm. If true, we will detect an edge at any time, being between the arm and background.

Thus,

(2) $$line(x)=\{\begin{array}{c} leftElbow+\frac{middleLeft-leftElbow}{times/2}*x,\;if\;x\leq times/2\\ middleLeft+\frac{leftShoulder-middleLeft}{times/2}*\left(x-\left(times/2\right)\right),\;\mathrm{else} \end{array}$$

To calculate the gradient at position x, we use the following Formula (3);

(3) gradient(x)=line(x+1)−line(x)

Line, describes the triangle function on the given point.

It returns the color in RGB format at the point. The difference between colors in RGB is defined as (4);

(4) $$difference(x,y)=\left|x_{R}-y_{R}\right|+\left|x_{G}-y_{G}\right|+\left|x_{B}-y_{B}\right|$$

We map the 3D RGB-space onto 1D space with this function, making it easier to compare two numbers and compute the gradient on an array with the size $size_{pixel}$ that represents all pixel values along the triangle function. The result of this computation is an array with the size, $size_{pixel}-1$. This array describes the difference between two neighboring pixels. By filtering this array for a value above a given threshold, which needs to be calculated or guessed, we know that there will be a considerable change in color. This has to be an edge in the image. At least on the color level, if there is an edge, we can conclude that the patient is wearing a shirt and the sleeve is not above the injection point. If the patient is wearing a shirt whose color is the same as his skin color, the edge would not be detected. The algorithm will evaluate this as an injection-free point. If all values are below the threshold, the patient is ready for injection; otherwise, he is not ready.

### 3.2. Injection Point Estimation

**Figure 5:**
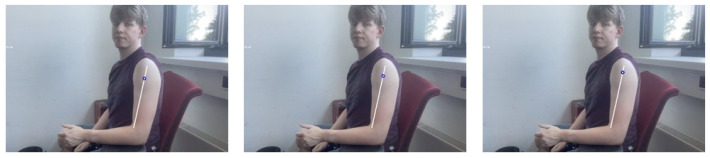
From left to right 75%, 80%, and 85%.

Complications associated with intramuscular injection can be abated if the right point is accurately predicted. Four common locations for inoculation are the arm (deltoid), upper outer posterior buttock (gluteus maximus)–also referred to as the dorsogluteal site, the thigh (vastus laterallis), and the lateral hip (gluteus medius)–also called the ventrogluteal site [31]. For convenience, tests will be limited to the deltoid muscle. The injection spot is in the middle of the deltoid muscle about 2.5 cm to 5 cm below the acromion process. In general, it is three fingers across the deltoid muscle and below the acromion process [32]–that is, three finger widths below the middle of the muscle. Computations were carried out to determine the ratio of arm length to distance to the injection point. We arrived at 80% and picked 5% variance for disparities in upper arm lengths. Three different videos were shown to nursing students to select the right estimation at 75%, 80%, and 85%, as shown in Figure 5. These medical students accepted the 80% estimate as having a better chance of spotting the injection point. Thus, we arrive at the following equation for determining the injection point (5);

(5) injectionPoint=line(ceil(times∗0.8))

### 3.3. Hand to Injection Point Mapping

The coordinates picked from images seen by the robot have to be interpreted in real world human dimensions. Here also, our approach is based on three assumptions:

1. The distance from robot to patient is predetermined.
2. The robot has a direct line view of the patient.
3. The injection point is below the head of the patient.

**Figure 6:**
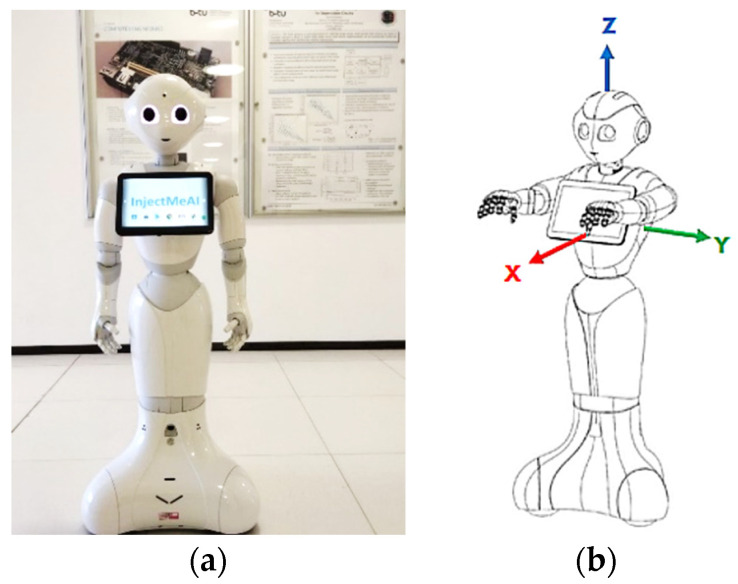
The Pepper Robot. (**a**) Front view displaying top-camera; (**b**) Robot coordinate system©, source [25].

These make it possible to consider the distance along the *x* axis only while keeping the *y* distance unchanged. Figure 6 shows the coordinate system on the Pepper robot.

Whereas the midpoint of the image is known in both real world and image coordinates, the injection point is known only in the image coordinates. Therefore, the task reduces to finding the real-world coordinates of the injection point on the patient. The first step is to look at the midpoint of the image and at the HeadFrame of the patient, which is in the middle of the image. The midpoint can be described as

$$MP(widthOfimage/2,heightOfimage/2)\mathrm{and}\;\mathrm{the}\;\mathrm{HeadFrame}\;HF(fixedDistnce,y_{h},z_{h}).$$

where $y_{h},z_{h}$ are coordinate axes on the human body.

**Figure 7:**
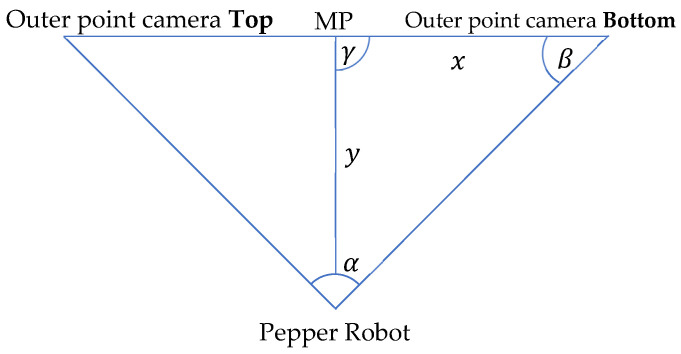
Theoretical limb movement visualization.

We want the frame position $F(x_{2},y_{2},z_{2})$ from a given Image Point $IP(x_{1},y_{1})$. The third assumption permits $y_{2}=y_{h}$. The next step is to map the y image coordinate to the z real world coordinate. We use the ratio of pixels to meters to accomplish this. It is known that the midpoint of the image is 3 m away from the robot (assumption 1). It is also known that the viewing angle of the whole image from the robot is 44.30 degrees, and that the distance between a surface and an object is calculated via the perpendicular. With this information, we re-present the problem using a simple triangle. The annotated triangle can be seen in Figure 7. It has the following properties: α=viewingAngle/2, y=3, γ=90deg, and *x* is unknown. We use Equation (6):

(6) x=(y∗sinα)/sinβ

If we insert the given values into the formula with β=180−α−γ=67.85deg, we get x=1.22 m. This means the distance from MP to P(1208,480), which is the middle point at the right side, in real-world dimensions is 1.22 m, and one pixel on that line is approximately 1.22/480 m. To compute the real-world coordinates for the z value, which corresponds to the direction of the value in the image, we need to calculate the change in the y dimension in relation to the middle point, then multiply it by the pixel-meter ratio. Moreover, the last step is to add it to the known z value of the middle point. We use the Formula (7) below to calculate the new *z* value;

(7) $$z_{2}=\left(\left(y_{h}-480\right)\times \frac{1.22}{480}\right)+z_{h}$$

The x value is estimated via average human sizes. Therefore, it is necessary to know the gender of the patient. If this is known, we use the 95% percentile as the shoulder width. For women, this value is 48.5 cm and for men it is 52.5 cm [33]. With these values and assumptions, we can create the following translation vector (8) from the HeadFrame:

(8) $$\upsilon_{tr}=\left[\begin{array}{c} shoulderWidth\\ 0\\ \left(\left(y_{h}-480\right)\times \frac{1.22}{480}\right)+z_{h} \end{array}\right]$$

### 3.4. Joint Angle Estimation

**Figure 8:**
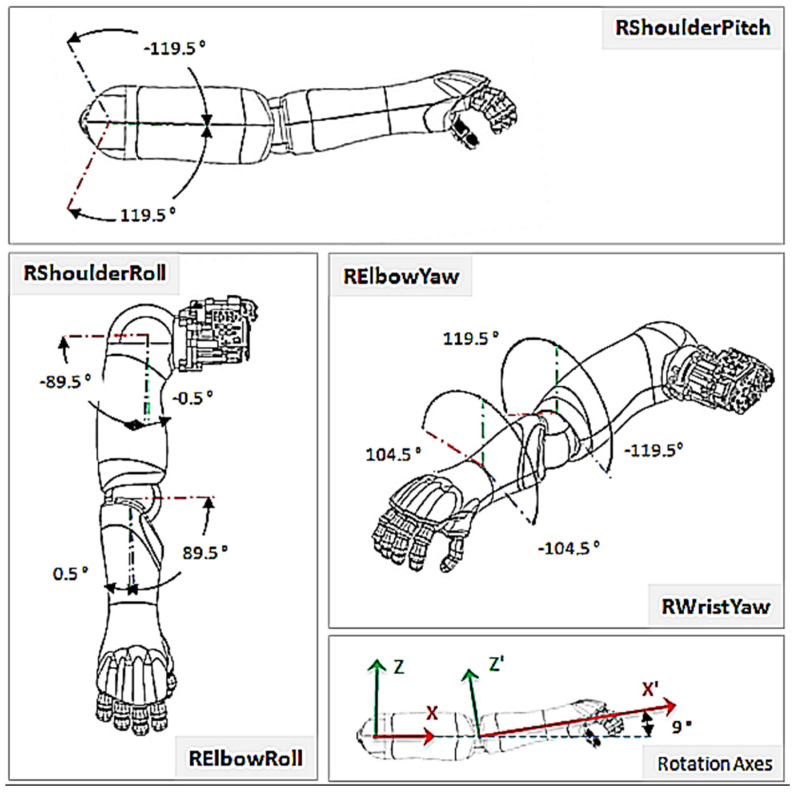
Right Shoulder Rotations ©, source [25].

The next task is to move the arm of the robot in such a way that the hand stretches to the correct injection point, theoretically. Two points are known at this juncture; where the robot’s shoulder starts which is the start of its arm, and where the patient’s injection point is. These are 3D coordinates that can be used for movement on the autonomous humanoid robot. To calculate the injection point position in the coordinate system from Pepper, we use the head as the starting position, consider the head to shoulder offset, and calculate the shoulder coordinates. We can calculate the vector from the shoulder point to the patient’s injection point when this is done. This can be done with Equation (9);

(9) $$\upsilon_{rp}=P_{sR}-P_{iPt}$$

where $\upsilon_{rp}$, is the resulting vector between the robot’s shoulder and the injection point. $P_{sR}$ describes the shoulder point of the robot in 3D coordinates while $P_{iPt}$ describes the injection point on patient, also in 3D coordinates. Two joints are present in each shoulder; shoulderRoll and shoulderPitch. The shoulderRoll handles rotation around the *x*-axis while the shoulderPitch handles rotation around the *y*-axis (Figure 8). However, the shoulderRoll does not need to be taken into context, since it would only adjust the injection angle and not the direction through which the hand reaches the patient. To perform a more human-like injection, the shoulderRoll is set to 0 degrees. This leads to the condition where the elbow stays below the shoulder, looking more like how a human will deliver the injection. We can again use a simple triangle (Figure 7) to describe the problem. The three points to be considered are: the shoulder point, the elbow point, and the injection point, which are all unknown. 

From manufacturer’s data [25], the upper arm length of Pepper is 181.20 mm and the lower arm is 150.00 mm. The distance remaining is the one between the shoulder and the injection points. However, because the vector is known, the distance can be easily calculated with the Formula (10);

(10) $$\left|\upsilon \right|=\sqrt{\upsilon_{x}^{2}+\upsilon_{y}^{2}+\upsilon_{z}^{2}}$$

**Figure 9:**
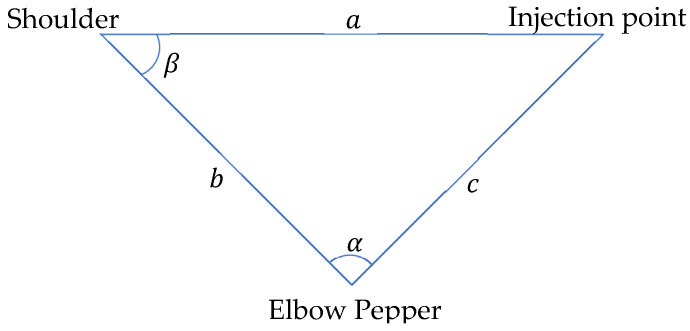
Theoretical Hand Movement Visualization.

With this information, we can compute the parameters in (Figure 9) as follows (11);

(11) $$\alpha =arc\cos\left(\frac{b^{2}+c^{2}-a^{2}}{2*b*c}\right)$$

$\begin{array}{l} a=\;\mathrm{distance}\;\mathrm{between}\;\mathrm{shoulder}\;\mathrm{of}\;\mathrm{robot}\;\mathrm{and}\;\mathrm{injection}\;\mathrm{point}\\ b=\;\mathrm{robot}’s\;\mathrm{upper}\;\mathrm{arm}\;\mathrm{length}\\ c=\;\mathrm{robot}’s\;\mathrm{lower}\;\mathrm{arm}\;\mathrm{length} \end{array}$

We use Equation (12) to calculate the angle at the shoulder;

(12) $$\beta =\arcsin\left(\frac{lengthLowerArm*\sin\alpha }{\left|\upsilon_{rp}\right|}\right)$$

Angle α will be used for setting the ElbowRoll(Figure 8) and β, the shoulderPitch. The ElbowPitch can be taken into consideration, but it is not necessary in this use case. This is because Pepper will move 10 cm aside from the injection point, implying that the ElbowPitch does not need to be adjusted.

## 4. Implementation and Evaluation

Application development on the autonomous humanoid robot has changed over the last few years [1]. Programs were coded and run directly on the NAOqi 2.5 operating system. Such codes had direct access to sensor data and could also control the robotic limbs. The programming languages primarily used were C and Python 2. Softbank’s introduction of the QiSDK on the Android Integrated Development Environment (IDE) has shifted control from a low-level to a high-level. On NAOqi 2.5, the robot (Pepper) was the main controller. It could control what appeared on the tablet and also move independently. With the QiSDK, roles have been reversed; the tablet is now the main controller. This leads to enhanced possibilities on the programming side. Java and Kotlin programming languages can also be used for coding. Softbank recommends the newer and safer Kotlin. The robot accesses the tablet over USB. The robot can rightly be described as an API that picks stimuli for the tablet. An added advantage derived from the transition from Choregraphe to Android is that it is now very easy to build seemingly complex applications to run on the robot. Almost all Android applications can be ported to it with ease, allowing the use of any feature that is supported by API version 23 and above. What needs to be added to it most importantly is the implementation of the RobotLifecycleCallbacks interface. This interface enables Android activities to get notified when the robot focuses on the program. The robot can only focus on one program at a time.

### 4.1. Implementation Concept

Our implementation was inspired by tutorials from Softbank and various repositories that worked with the QiSDK [34,35,36]. Some abstract functions had to be implemented from the *RobotLifecycleCallbacks* [37]. The compulsory functions are *onCreate*, * onDestroy*, * onRobotFocusGained*, * onRobotFocusLost* and *onRobotFocusRefused*. The *onCreate* function can be regarded as the constructor for the application. *onFocusGained* is called when the robot focuses on the application. At this point the implementation will be called and started. If this is carried out in *onCreate*, the robot may focus on a different activity (in the program) and thereby lose the tablet’s connection to Pepper, resulting in an error state. The application starts with the press of a button. This button is activated only when the program is ready to run and the Pepper robot is focused on the application. The detection function begins when the button is pressed. Due to the usage of threads, the detection is wrapped in the *runBlocking* function [38]. This function runs a new coroutine and blocks the current thread until execution completes. To begin human pose detection, it is necessary to locate the closest person in the vicinity of the robot. When a patient is found, we need to estimate the pose of this person. It is a requirement by BlazePose to obtain a face in the input image. To achieve this, the robot moves three meters away from the patient and takes a snapshot. It lowers its head and focuses on the patient’s chest, getting a good shot in the camera with less free space and ignoring body parts. The resulting image is piped into the pose estimation and detection algorithm. If the person is standing, he is asked to take a seat. When a person with the right pose is found, the bare shoulder detection starts. When the shoulder is bare as expected, the robot moves closer to the patient and raises its arm to the height of the injection point. At this stage, the injection hardware system will be activated. 

### 4.2. Closest Human (Patient) Detection

When more than one person is near Pepper, it should choose the nearest person as the patient. Pepper has the built-in high-level function to detect human objects. This object holds information about the position of the head, age, gender, and excitement state. To obtain the closest person, the *HeadFrame* is of interest. To locate the patient, we take all human objects in the field of view of the robot and calculate the distance from each. This information can be obtained by calculating the translation between the *robotFrame* and the *HeadFrame* of the current person. The distance metric is defined as $\sqrt{x_{tr}^{2}+y_{tr}^{2}}$, where *tr* is the translation. The next step is to move three meters away from the patient. To calculate the translation from the *robotFrame* to the target frame, *fromXTranslation* can be used. The axis is relative to the robot, not to the human (Figure 6). This means that Pepper will be aligned with the patient’s body. To obtain the right frame, the *HeadFrame* is used at *x_tr_* from three meters. This frame has to be an *attachedFrame*, otherwise this frame will move with the movement of the robot which is not the expected behavior. Now that the frame to which the robot should move is calculated, it needs to move to this point. This can be achieved in two different ways. One of two functions provided by QiSDK can be used, *GoTo* or the *StubbornGoTo* [39]. They are different in implementation and being open-sourced can be refined as needed. Based on this, the *StubbornGoTo* has been applied with some changes. The robot will only move in straight lines and try two times to move to the target frame. This is necessary because the robot has problems differentiating shadows from walls. Unfavorable lighting conditions can also lead to unpleasant situations. At the end of the movement, Pepper should look at the patient. This is achieved by calling the built-in *LookAt* function to obtain the *HeadFrame* of the patient. The last step is to take a snap shot of the patient. Therefore, the *TakePictureBuilder* is used. It uses the top camera of the robot, which is situated on its forehead as shown in (Figure 6). This returns a *TimestampedImage* that is converted into a *bitmap* image for further processing.

### 4.3. Pose Classification

There are two variants of BlazePose that can be employed—*lite* and *full* versions. Their main differences lie in the computation time and accuracy. The single image mode takes more computation time but is also more accurate than the video mode. The single image mode is used because the computation time is only about one second, which is fine. The *bitmap* resulting from the picture needs to be converted into an *inputImage*. An image of type *inputImage* is used for interacting with Google’s vision API and provides different encoding functions when fed into the network. First, the pose estimation algorithm is called using the function *poseDetectorImage*.*process*. This returns a task which is used in asynchronous programming. In other words, we can decide what to do with the output after the function completes the computation. We pick the detected pose key points (33 in total) and pipe them into the *poseClassifierProcessor*. This returns the pose that is detected with its accuracy. The classifier is a simple k-Nearest Neighbor (k-NN) classifier. At the end, this result and pose key points are combined in the *PoseWithClassification* class to generate the final result.

### 4.4. Training Data

A base dataset [40] and a custom dataset, covering students posing for injection at The Brandenburg University of Technology, were used for training. The training data were stored in a CSV file with the following structure: image file name, class, key points. The classes being: sitting, lying, and standing. Videos from these classes were split into frames and fed into the classification CSV generator from Google [41]. The training was performed in a Docker container on a local machine and not on the Colab Cloud servers. Working on the Colab Cloud was not helpful because the computation resources were taken away after sometime, resulting in multiple failed tries. The Docker container had the exact configuration as the Colab Cloud. This led to easy usage of the container without adjustment.

### 4.5. Bare Shoulder Classification and Injection Point Spotting

The estimated key points are revisited to obtain information on shoulder and elbow key point pixel locations. The pixel from shoulder to elbow is needed. An iterative function is used for this. Making use of a while loop, it starts at zero and runs until the variable *times* is encountered. The values are then pushed into a dynamic array. There are different ways to determine the difference between the current entry in the list and its successor. Here, the *zipWithNext* function is used with the zip function *colorDiff*. After this, a color gradient is obtained to check if any value in the array is above a specified threshold. To test this, the *all* function is used. If all values are below the threshold, we proceed with the injection point estimation. This is done by using the same function as in the bare shoulder classifier. In pixel values, the injection point is calculated using x=times*0.80. This produces the point that was seen as good for injection by the medical students. The translation is created by the *TransformBuilder* with a translation from a 3D vector. The x value is chosen based on the gender of the person that should get the injection. This information is available in the *human object* class. For safety reasons, 3 cm are added so that Pepper does not move extremely close to the patient. The z coordinate is calculated by inserting the $y_{iPt}$ for $y_{h}$. In addition, the resulting z translation needs to be multiplied by −1. This has to be done because the image coordinate is rising from the top to bottom. However, the z coordinate has its zero at the ground. This means that if the pixel position is increasing, the z value needs to decrease. In contrast, to the earlier assumption, the *y* value is not set to zero but to 0.14974, the y offset from Pepper’s shoulder relative to its head. This has to be done so that the robot’s arm is before the injection point and not its head. As this point should not move when Pepper is moving, it is attached to the *HeadFrame* of the patient. Hand raising is performed by moving the right arm joints per calculated angles, so that the robot’s hand arrives at the height of the spotted injection point.

### 4.6. Joint Movement Actualization

Design characteristics place the upper arm length of the robot at 18.120 cm, its lower arm at 15.0 cm, and its hand at 7 cm long. We can therefore confirm that the robot can be at most 18.120+15.0+7=40.120 cm away from the patient to reach the injection spot. A shorter distance is more appropriate. When planning its movement, the robot prefers a clear view around obstacles from a distance of 120 cm [42]. We can again use the custom *StubbornGoToBuilder* because it is just moving in a straight line and mostly ignores obstacles. In the end, the body of Pepper is positioned facing the point towards which it should move. It makes two attempts to reach the point. The first attempt is to get as close as possible to the frame. The second attempt corrects any drift while moving along the 3 m distance to the patient, sets the maximum speed to a value that looks very safe to the patient, and stops Pepper from moving too far when it does not brake hard enough. Continuing the process, it looks at the injection point that has been calculated. This is needed to compute the joints’ movement. To start this, it computes the distance between the shoulder of the robot and the injection point. The offset for the shoulder is supplied by the *HeadFrame* of the robot. To access the information, the *gazeFrame* is used with added values from the shoulder, defined in [25]. The *distance* function can be used to obtain the distance from the newly calculated frame to the injection frame. This returns the distance in meters. Now all values are available to compute the α and β angles. The α value requires some adjustment when being used for the joint movement. This is because when setting the *elbowPitch* to zero degrees, it is in reality 180 degrees. When we want to have a 120-degree angle, we move the elbow by 60 degrees. This places the real value of α outside the computation at 180−α. Pepper can now perform small movements at the *shoulderRoll*. If this adjustment is not made, Pepper will move very slowly in order not to hit the tablet in any way. The allowable restrictions, extracted from [25], are shown in Table 3. Having completed the computations, the Python Wrapper function can be called. The parameters needed are the joint names, the calculated angles, the translation time, and if absolute angles are given or not, and whether movements are performed asynchronously or otherwise.

### 4.7. Python Wrapper

Currently, the tablet does not support direct access to sensor data or the inner system of the robot [43]. It is however, possible to use the NAOqi 2.5 on the robot. This permits the tablet *Secure Shell (SSH)* to be loaded onto the robot. From this point, it is possible to control the joints of the robot [44]. This is where a Python wrapper comes in handy. The resulting strings will then be executed on the robot via *SSH*. A library for enabling *SSH* on the tablet *sshj (SSHv2 library for java)* from Hierynomus was used [45]. When joint movement is needed, the wrapper connects to the robot and offers SSH connection that can be used to execute Python code. The IP address is from the USB interface and not from *WIFI*. This has the advantage that it is not affected by changing the network. The IP address is obtained by establishing a connection via *SSH* to the robot and running *ipconfig* [46]. When a connection is established, the wrapper creates an environment for working with the Python SDK. This results in initialization of all services that are needed to perform the desired actions. Therefore, a function, *createEnv* was made. To move the joints on Pepper, the *ALMotion* service is needed. It is responsible for everything connected with movement from current joint’s position to the interpolated joint angles. This service can be obtained from a *qi application session.* Qi applications are programs needing resources from the robot running NAOqi. The Python Wrapper functions as the Python SDK. Therefore, it sends parameters over the SSH connection that has been established. Presently, the supported functions are *angleInterpolation* and *setStiffness.* However, needed functions can be easily added to the wrapper. With the aid of the wrapper, it is now possible to perform computations using libraries in Kotlin and use Python for directing the joints.

### 4.8. Autonomous Behavior

Pepper is imbued with an emotional engine that supports its autonomous abilities to act human-like. Under these abilities is face detection, where it looks in its idle state, into the patient’s eyes, emulates human gestures, and uses its in-built text-to-speech API. The *sayBuilder* is used to interact with the API. When performing critical tasks like taking a snapshot after looking at a given frame, the autonomous abilities are disabled to stop unwanted movements. Such movements can be considered as safety hazards when working on patients. Hence, these autonomous abilities are disabled for a short period only, with a *holdAbilities* function. If this is done long term, the humanoid robot will lose its human-like characteristics. 

### 4.9. Findings and Discussion

**Figure 10:**
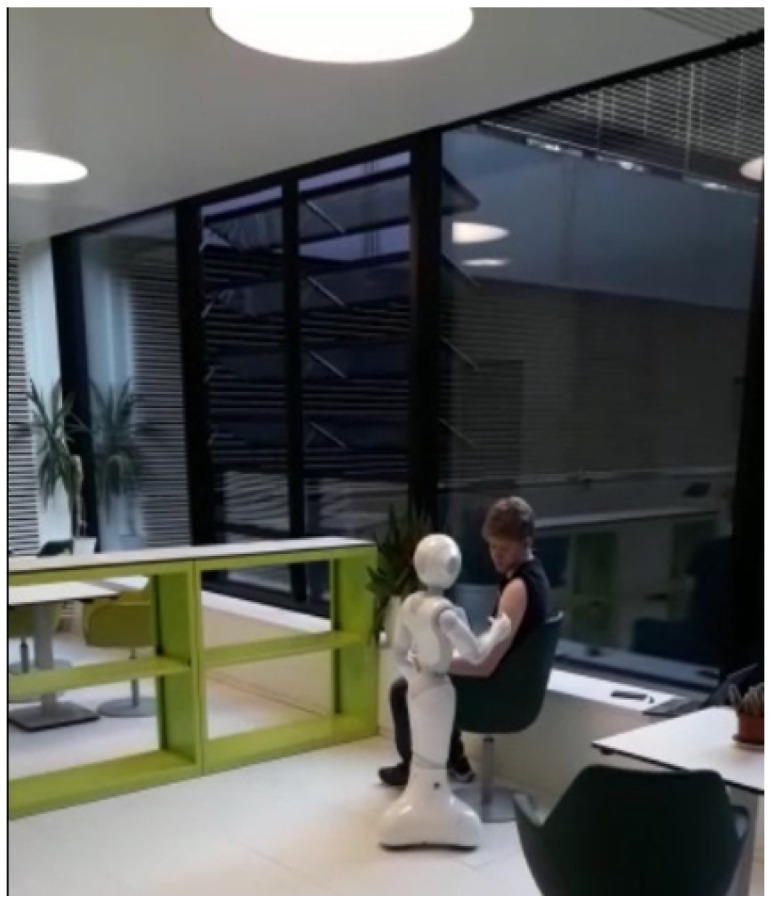
Software Module Evaluation.

Tests were mainly carried out on adult males at the chair of computer engineering, Brandenburg University of Technology, under varying lighting conditions. The robot has an arm reach of 40.120 cm. The upper arm lengths of our test subjects ranged between 23 and 32 cm. At no time did the robot cause harm (injury) to any of the testing participants. The patient’s seating was also rotated from a transparent glass background to an opaque wall (Figure 10); Pepper itself needs good lighting conditions to work correctly. The bare shoulder detection exposed a discrepancy where some combinations of skin color and shirt color were not supported under certain lighting conditions. This discrepancy can be addressed through further training. The patient was required to wear short sleeves irrespective of the ambient temperature conditions; an avoidable discomfort. The robot reached the injection spot in under 60 s in real time. However, after several trials we can confirm a success rate of about 80%. A change in seating position after pose detection and classification resulted in a change in direction of the robot’s heading. Thus, the robot could not reach the injection spot. Further testing is needed as unpredictable delays in joint movements occur occasionally when Pepper is interacting with patients. Such movements must be detected for the robot to call for assistance from medical staff during peak service hours. To enhance real time computation and accuracy, key points that are not needed at particular stages in the program can be selectively ignored. Another point to consider is the use of Pepper’s depth sensor data to support injection point spotting. This could increase injection point estimation accuracy on the deltoid muscle. Compared to the stationary robotic arm introduced by the University of Waterloo [47] for vaccinations in upright positions, our mobile robot supported with an emotion engine can interact with humans verbally and perform the injection point estimation on patients in more comfortable positions, without direct inputs from the patients. Thus, our system also eliminates the need for patient training. As progress is made in this area of research, it is hoped that benchmark data for evaluating the performance of autonomous vaccination robots will become publicly available.

## 5. Conclusions

A software module for vaccination at hospitals and medical centers has been developed and tested on an autonomous humanoid Pepper robot. The robot can interact with patients verbally and identify their readiness for inoculation. When confirmed, it checks if the bare shoulder requirement is met, draws closer to the patient, and moves its joints in the correct sequence to reach the injection spot. Tests carried out have confirmed the success of our research with 80% positive results, all occurring in under 60 s of real time. No injuries were recorded during testing. For the first time, we have shown how a humanoid robot can be engaged in the vaccination process at hospitals and medical centers in order to save lives by reducing human-to-human contagion. In the near future, this proof of concept can be used to develop efficient ways of working and interacting with infectious patients in hospitals and assisted living spaces, especially during pandemics.

## Figures and Tables

**Table 2:** Single Instance Run per Pose Estimation Algorithm.

Pose Estimation Algorithm	Single Instance Run (s)
BlazePose	0.0086
Soft gated skip connections	12.375
OpenPose	200.45

**Table 3:** Movement Restrictions Imposed on the Right Arm of Pepper.

RElbowYaw (^o^)	RElbowRoll min (^o^)	RElbowRoll Max (^o^)
−119.5	0.5	83.0
−99.5	0.5	89.5
0	0.5	89.5
60.0	0.5	78.0
119.5	0.5	78.0

## Data Availability

Not applicable.
